# Community case study: overcoming barriers to social participation for improved local health governance in Madagascar

**DOI:** 10.3389/fpubh.2026.1778275

**Published:** 2026-09-08

**Authors:** Dörte Petit, Dheepa Rajan, Kira Koch, Charlotte Poulussen, Jean de Dieu Marie Rakotomanga, Marc Rajaonarison, Csongor Bajnóczki, Sarah Brousse, Hyppolite Kalambay, Saina Isiraely Rambinintsoanomenjanahary, Katja Rohrer-Herold

**Affiliations:** 1 WHO, Geneva, Switzerland; 2 European Observatory on Health Systems and Policies/ WHO European Centre for Health Policy, Brussels, Belgium; 3 National Institute of Public and Community Health, Antananarivo, Madagascar; 4 Permanent Mission of the Republic of Madagascar, Geneva, Switzerland; 5 Action Against Hunger, Paris, France; 6 WHO Regional Office for Africa, Brazzaville, Republic of Congo; 7 WHO Country Office, Antananarivo, Madagascar

**Keywords:** Africa, community engagement, health governance, policy making, social participation

## Abstract

Government engagement with communities are critical for achieving health equity goals. The available literature is plentiful on community and civil society’s barriers to meaningful participation. However, considerably less documentation abounds on *how* to address barriers to equal interaction within the participatory process, with concrete interventions tested and assessed. This paper aims to bridge that research gap by studying community engagement for health policymaking in Madagascar, focusing on how policy makers can design interaction with communities in a way which decreases power imbalances within a participatory space, thereby enabling constructive dialogue to address health systems issues. We conducted 37 key informant interviews across all levels of the health system (community, district, regional, and national) in one urban and one rural district of Madagascar’s Atsimo-Andrefana region. Purposive sampling techniques combined with emergent, opportunistic sampling, were used based on participants’ experience with the country’s national health planning processes. Multiple rapid literature reviews – before, during, and after the interview phase - helped to guide, steer, and underpin the main conclusions presented in this article. This paper points to three simple strategies which can contribute to providing counter-vailing power to those with less voice: using the same language as those whose views and input are solicited; creating smaller, homogenous dialogue groups with participants whose voices are less heard; and bringing consultations to people’s own environment. While these strategies are neither novel nor complicated, they are rarely leveraged by those seeking people’s views on health policies. It is hoped that by providing qualitative evidence suggesting the effectiveness of these strategies, when properly implemented, they will become more widely used to promote meaningful community participation for health policymaking.

## Introduction

Social participation is the systematic involvement of people, communities, and civil society in health decision-making. Governments that promote and institutionalize social participation are better positioned to strengthen governance and advance health equity (1–4).

To achieve it, health stewards must invest in mechanisms that regularly incorporate people’s voices into health planning and policymaking, a task that remains challenging in practice (5, 6).

Power imbalances in participation can occur when some stakeholders have more influence or decision-making power than others. This can lead to certain groups being marginalized or excluded from decision-making processes and can result in health inequalities. Hence, one of the facets of that complexity is that meaningful social participation is fostered when participants are capable to express themselves freely. Achieving this, however, requires at least an enabling micro-environment within the participatory space, and ideally, a broader enabling environment within the health sector and society at large (7). Many participants, especially those whose voices are commonly less heard, have unequal starting points and face socio-cultural and economic barriers to participation which can be disempowering. At the same time, most policy-makers struggle with the ‘how’ of managing a complex participatory process (7, 8). This ‘how’ involves creating a micro-environment within the participatory space where all stakeholders are on as level a playing field as possible to express themselves without fear of reprisal (7).

The literature on the use of participatory mechanisms specifically for formulation and decision-making of health policies in the African region is scarce. Studies which do explore the extent of local stakeholder engagement in health policy processes tend to examine barriers to meaningful engagement, with little analysis on practical solutions to tackling these barriers (9–12).

Participation has been researched throughout the African region (though less so in Madagascar particularly) as a method to support and improve health programme implementation. Existing studies largely use participatory methods to improve access to health service delivery (9, 13–17), or to support health research purposes (18–20). The Madagascar-specific literature on participation is mostly focused on the environment sector (21–23), and water and sanitation measures (23, 24).

## Rationale

Governments often fail to address the power imbalances that undermine meaningful participation, despite their commitment to participatory health policymaking. Research has largely overlooked practical strategies for creating participatory spaces where stakeholders can contribute on equal terms.

This article aims to bridge the evidence gap, while taking the complex social and political context of Madagascar into account: The purpose is to understand how to overcome barriers to social participation for improved local health governance in Madagascar, but also beyond, as many of the above-mentioned barriers to participation are not unique to Madagascar (7, 25–28). There are large gaps in the literature relating specifically to *operationalization* of participatory mechanisms within the decision-making process, beyond the study of barriers to participation. Therefore, this study examines how governments can strengthen their capacity to organize participatory spaces that address barriers to meaningful participation. Specifically, it addresses the following research questions:

- How can governments design participatory spaces that promote equitable participation?
- Which participatory formats are most effective for ensuring that stakeholders can contribute on equal terms?
- How can participatory processes address equity concerns and existing power imbalances among stakeholders?

By answering these questions, this study seeks to identify practical strategies that can strengthen participatory health governance in Madagascar and inform similar efforts in other African settings and beyond.

## Objectives

This study aimed at addressing two interrelated objectives:

1. To identify and examine the socio-cultural and economic barriers to community participation to establish an empirical basis for developing targeted strategies to address them.
2. To determine which community engagement interventions, hold the greatest potential for rebalancing power relations between communities and local health authorities, thereby lowering barriers to participation and facilitating meaningful, equitable interaction.

For better understanding of the setting, we provide an overview of the Malagasy health system in Box 1.

Box 1 **Brief overview of the Malagasy health system.**

The Malagasy health system is organized into a pyramid of four levels, each representing a platform for health service delivery: central, intermediate (regional), district (peripheral) and community level. The community level includes Community Health Workers (CHWs) who primarily engage in health promotion. ‘Basic health centres’ (*centres de santé de base* or CSBs) are also part of this level, with a network of 2,600 CSBs serving as the first point of contact between the community and the health system.

Community health services were explicitly recognized as pivotal for the country’s overall development in the 2009 National Community Health Policy (29) (updated in 2016). Here, communities are considered not only beneficiaries but also active stakeholders integral to health sector functioning. The Policy bolstered support for Fokontany (village) and Commune Health Committees with CHWs as key members.

Madagascar’s predominantly rural geography means that most health facilities beyond the CSBs are far away for Fokontany communities. Besides this geographic access barrier, secondary and tertiary care facilities lack adequate personnel and resources, leading to health service under-utilization and increased use of traditional medicine (30).

Box 1 provides an overview of the Malagasy health system.

## Methodology

Between 2019 and 2023 the above-described objectives were addressed in two ways:

1. We studied targeted interventions to include community voices in district health strategic planning and monitoring in two Malagasy districts. These efforts were undertaken with support from an international NGO (Action Against Hunger – ACF in its French acronym) in collaboration with a local NGO (Social and Health Action Relief Organisation – ASOS in its French acronym) and is described further in the following section and in Box 2. To do this, we conducted key informant interviews where the NGOs were active with its community participation efforts.
2. In parallel, we conducted a literature review about the topic specifically on Madagascar and the broader African region as well as the activities of the NGO.

Box 2 **Key informant interviews.**

A semi-structured interview guide was formulated based on the following themes:

- National health planning and decision-making in the health sector, including the role of the community within those processes.
- Community contribution to national health planning through the targeted strengthening of community voice at local level.
- Bottlenecks impeding community participation in health planning processes.
- Factors that encourage community participation and engagement.
- Community structure and organization which encourages involvement in health planning processes.

Interviews touched upon community participation in general as well as targeted local community engagement efforts.

**Figure 1:**
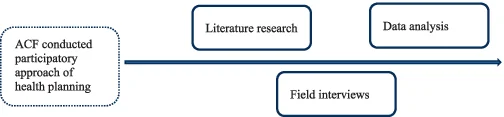
Flow chart of the methodology.

Figure 1 provides an overview of the methodology:

This study adopts a qualitative case study design of an NGO-supported participatory intervention embedded within routine district health planning processes. Action Against Hunger (ACF) acted as a facilitator of participatory workshops in collaboration with local stakeholders, providing the context within which this research was conducted, rather than being the object of evaluation. Sampling was purposive and opportunistic, focusing on individuals directly involved in or knowledgeable about the intervention and national planning processes; as such, the aim was depth of insight rather than representativeness.

In the following, we provide some background, describe the intervention conducted by ACF, the literature analysis and then the qualitative interviews.

### Socio-cultural challenges to social participation in Madagascar

Research on social participation in Madagascar, especially in health, is limited. Available evidence indicates that entrenched social hierarchies constrain meaningful participation. Political and economic elites have historically maintained their privileges, while rural populations have remained largely excluded from decision-making processes and have had little influence over public affairs (31–33).

In the 2024 Afrobarometer survey in Madagascar, roughly 43 percent of respondents from a representative sample said they do not feel “very free” or “free at all” to express their opinions; this number has been steadily increasing since 2009 when the percentage share was 22% (34, 35). 42% of those surveyed were unable to give an opinion on the level of democracy in their country, pointing to either overall low education levels or a lack of priority given to democratic expression. 57% of respondents agreed with the statement “people are like children, and the government should take care of them like a parent,” reflecting respect for hierarchy and the expected patriarchal role of those in higher civil servant positions (36). Even if nearly 55% of ordinary citizens thought that the condition of the poor should be improved, only 9% thought the poor should have a greater say in government decisions—an increase from 7.4% in 2009, but still very low (36).

Open confrontation is very rare in Malagasy society. Lack of confrontation, respect for traditional status and low expectations of accountability from leadership, combined with a fragile social structure, all reduce communities’ capacity for opposition and expression (33, 37).

### Amplifying community voice in local health strategic planning in Madagascar: an NGO-supported intervention

Increasing community participation in local health strategic planning is an explicit objective of the NGO support to existing government-led district processes, starting with a participatory situation assessment of the local health system, followed by a strategic planning phase (Box 3). The process is implemented via participatory workshops with a variety of stakeholders – regional and district health authorities, local and international partners, community health workers, and community members – where current challenges and potential solutions are collectively discussed. The workshops are meant to support district health authorities in developing and implementing a district health plan with the core principles of dialogue and participation (38–40).

Box 3 **An NGO-led approach to increasing community participation at district level.**

The NGO-led community participation approach comprises a number of steps, each of which are undertaken with local health authorities: preparation, primary and secondary data collection, then two assessment workshops to guide programming and planning (40). During the preparation phase, all key health stakeholders in the region and the districts in question are identified and mobilized, and a steering committee is created for local ownership.

After data collection, bilingual French-Malagasy assessment workshops are organized with steering committee members, ministry of public health representatives, regional & district leaders, administrative authorities, CSB heads and CHWs, community members, and local & international partners. The aim of the assessment workshop is to discuss data analysis results and assess local health system performance.

Based on the outcomes of the assessment workshop, a planning workshop is held, where a discussion takes place on possible solutions to on-going challenges. Workshop participants then prioritize district-level activities to meet the community’s immediate needs with a view to strengthening the local health system in the long term.

The planning workshop is followed by meetings with donors, civil society organizations, and national and regional public health directors to seek buy-in and financial support.

### Rapid literature review to guide primary data collection

To identify peer reviewed articles on social participation in health policy-making, a rapid scoping review was conducted based on a combination of the following search terms: community engagement, stakeholder engagement, social participation, participatory governance and health policy. Two databases, Scopus and Pubmed, were searched for articles in French and English published between 2000 and 2023 specifically on Madagascar and the broader African region.

**Figure 2:**
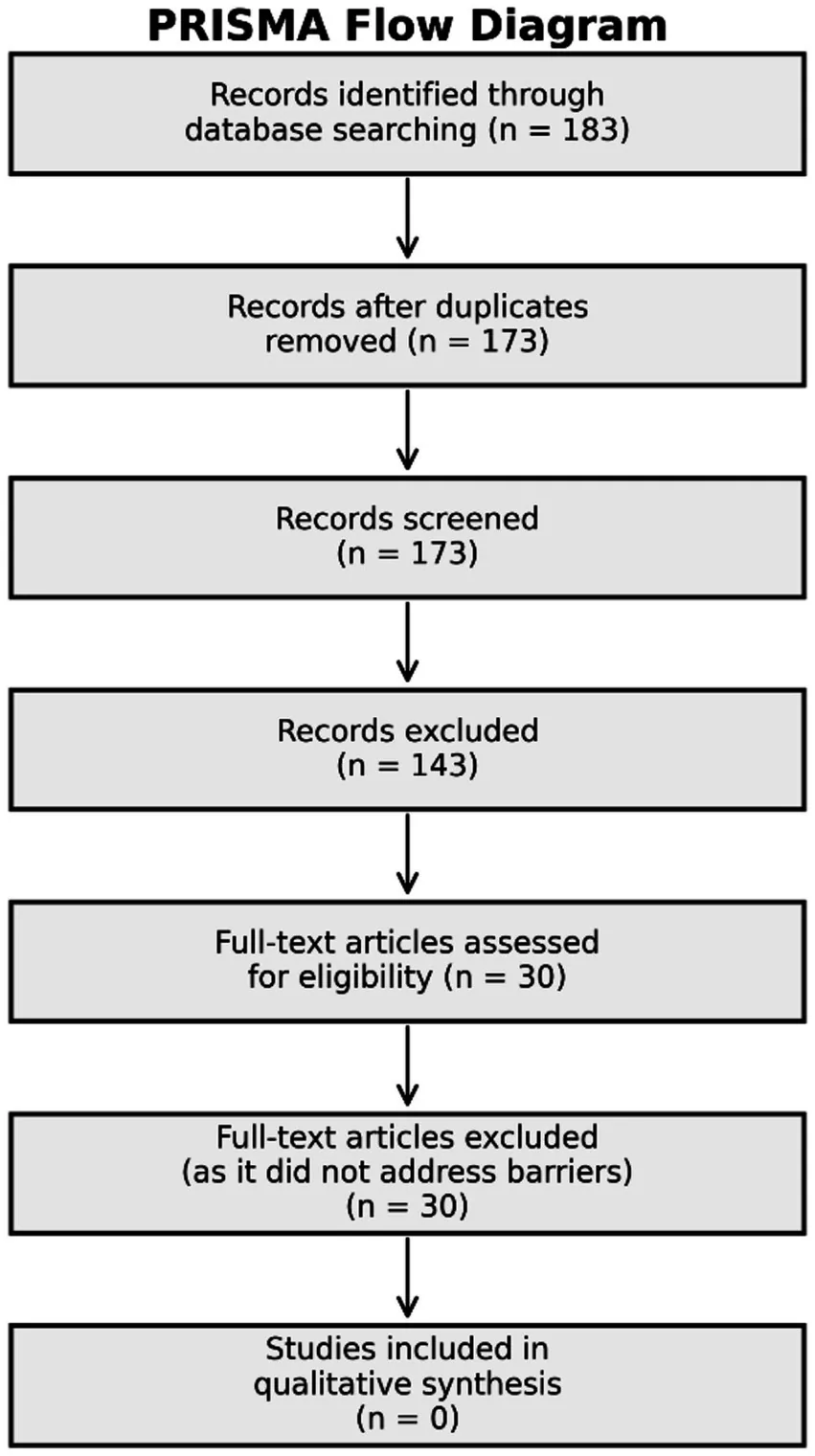
Rapid scoping review: results.

Hundred and eighty-three records were identified (see Figure 2). After removing duplicates, 173 records were screened, of which 30 were considered for full-text review. More than 50% of the records identified were published in the last five search years,^1^ which showcases a recent increase of interest in social participation. Articles were narrowed further down based on abstracts relating to participation for health decision-making, leaving 30 articles which were considered for full text review. Within this body of evidence on participation (12, 17, 41–43), no articles on addressing barriers, i.e., *how* to tackle power imbalances within the participatory space, were identified.

Given the lack of relevant peer-reviewed publications on our topic of interest, a rapid grey literature document review was conducted of 25 documents specific to the Malagasy health system and community participation (documents were all in French. This document review was also used to support the design of the semi-structured interview guides).

Given the scarcity of literature, primary data was collected within the remit of WHO’s programmatic work on social participation. Being part of a series of several WHO case studies, a semi-structured interview guide was developed for 9 countries, all of the case studies aimed to understand how people’s voices were brought into policy-making processes (7).

This case study was based on qualitative interviews. The guide was pre-tested in previous case studies and adapted for Madagascar based on insights from the above-mentioned targeted grey literature review as well as discussions with the WHO Madagascar Country Office and local and international NGO staff (see Box 3).

37 key informant interviews were conducted until data saturation by the principal investigator. The sampling process respected the following criteria:

1. In one urban and one rural district in the Atsimo-Andrefana region (Toliara 1 and 2 respectively) where the NGOs were active with its community participation efforts as mentioned in Box 2.
2. Care was taken to ensure interviewees came from different levels of the health system, from the community to the central level (43).

Table 1 shows the type of interview partners by health system level, highlighting the larger share of interviewees from the community and district level vis à vis the regional and national level. This was because only few people at the regional and national level had knowledge of the community participation intervention in Atsimo-Andrefana (as per Box 2) which was one of the criteria used to select regional/national health system level interviewees. However, for the purposes of this study, it seemed particularly important to hear the voices of the community and district levels.

**Table 1:** Interviewees by level.

Community and district level	Regional administration and civil society organization staff based at regional level	Central level
Heads of basic health centers, Heads of community- and *fokontany*-level health committees Medical inspectors from both districts Representative of the Toliara 2 district administration Total: 24	Projects focal point, ministry of health ASOS inter-regional coordinators (one contacted by phone from Toliara 1 and one from Toliara 2) ACF representative Representative from the regional umbrella platform for civil society organizations Representative from Tafatafa, a Malagasy civil society organization Regional focal point for HINA Atsimo-Andrefana, a Malagasy civil society organization Total: 7	Deputy technical director, Ministry of Health Head of the information systems service, Ministry of Health Director of prevention and community health, Ministry of Health ASOS headquarter staff WHO Madagascar representative National Institute of Public and Community Health representatives Total: 6

The principal investigator spoke French but not Malagasy; French-Malagasy translations were undertaken by local NGO staff members if the interviewee did not speak French. Each interview took between 45 and 60 min. Purposive sampling techniques combined with emergent, opportunistic sampling were used with the following key informant selection criteria:

- participation in key events as described in Box 3; or
- experience with the country’s national health planning processes.

The interviews were recorded with consent. Where consent for recording was not given, notes were taken. All recorded interviews were transcribed verbatim in French or Malagasy, the latter were then translated into French.

Each interview was coded by two out of a group of five researchers. To mitigate confirmation bias, the researcher group had mixed WHO and Malagasy institutional affiliations and additionally included an external person who had not been involved in data collection nor was familiar with the country context. All five co-developed a coding framework based on major emerging themes from the data set. Initially developed themes were examined closely for possible inter-theme connections and relationships (44), which led to a refinement of themes by linking and grouping them in an iterative process (45). This was followed by a 4.5-day workshop with the researchers who had coded the data plus one additional Malagasy health expert. At the workshop, thematic codes were triangulated with each other to enhance validity. Reliability was also increased through the iterative process of comparison, triangulation and revision of the coding framework (46).

## Results and findings

The records which were identified through the review of literature largely cover the use of participatory methods *for purposes of improving health service delivery*, including malaria control (47–49), HIV and AIDS (50–52), tuberculosis prevention (53) and reproductive health (54–56). In comparison, only few records discuss the inclusion of participatory methods within the health decision-making process itself (12, 42).

The analysis of literature also showed that participation is increasingly recognized as beneficial to implementing health policies, but evidence on the ‘how’ of setting up and sustaining participatory spaces for health decision making that provide opportunities for equal interaction and reduce power imbalances are under-researched. Thus, this results section is based exclusively ton findings from the interviews.

### Study objective (a): understanding the specificities of socio-cultural and economic barriers for meaningful social participation

This section examines the sociocultural and economic barriers (i.e., discrimination based on people’s sociocultural and economic status in society, e.g., ethnicity, income, living situation, age, gender, religious affiliation) identified in this study which hampered communities’ meaningful participation in the district planning workshops mentioned in Box 3.

### Fear of speaking up due to socio-cultural hierarchies

The interviews highlighted fear of people in power, or perceived to have power, as a factor which seemed to prevent communities from speaking up:

*Civil society representative: But there… there are CHWs who are a bit… a bit reserved, who did not talk much, either because, well, I do not know… a little self-conscious saying: “Yes, but my level… CHW level…*”*. Now, there are some in the meetings who do not say a lot of things… Now, that’s, I guess, it’s all about complexes or ... They are in front of people, in front of doctors, in front of midwives and all that, in front of everybody like that, well. Put someone who is in the bush, who comes there to chat with people who are a little higher than them. Well, it’s normal that they have complexes.*

Almost all interviewees expressed that people, especially those living remotely, are afraid of retaliation if they complain openly. Several interviewees cited health workers’ power over local people and their health as a factor that stopped them from complaining about issues such as quality of care or corruption. Interviewees at all levels of the health system shared this perception, indicating that this is a well-known phenomenon.

*National MoH cadre: Community agents work in the Fokontanys [villages] served by the CSBs. And if ever there are cases of illness in their family… [] if one complains at the level of the CSBs maybe there will be consequences because the CSB chief would not want to take care of their family… [] better not to speak than to be rejected by the CSB chief or by someone... Here, health is really life.*

*Community Health Worker (CHW): We tell them not to be afraid, but they fear for their lives because they are doctors in front of them, “just one prick can kill us.”*

*Regional government authority: it’s almost tradition. There is fear of vazaha [foreigners], fear of blowguns, that’s how it is. So there is fear of the consequences of the complaint, one is afraid of persecution afterwards, of not being provided care, like that. So people keep to themselves to avoid accusations of being a disruptor.*

These hierarchical constraints fostering a fear of power hindered frank discussion on sensitive issues. CSO and government (regional and central) interviewees indicated that uncontroversial topics such as fair distribution of bed nets or long distances to the health clinic are openly discussed while sensitive issues such as corruption and user fees are never brought up openly, especially in front of people in higher social and professional positions, such as the CSB head.

*National MoH cadre: Civil servants are important people, [communities] say. So they […] dare not complain directly about their establishments.*

*Regional government authority: Believe me, a farmer will never go to see the district head or the mayor because a health worker demanded this or that amount to deliver his wife’s baby, because… no. No, for them, it’s wiser just to pay up and not say a thing about it to anyone, or keep their mouths shut and leave, not go there because they do not have what it costs.*

In practice, this unwillingness to enter frank discussions on sensitive issues translated into lackluster participation from community members, CHWs, and also some heads of CSBs at workshops where attendance included their superiors in the health hierarchy. Many interviewees confirmed that participant reluctance to engage more actively was driven by the presence of this hierarchy.

### Cultural trait of keeping quiet

In addition to not speaking out due to fear, several interviewees pointed to people’s preference to keep quiet and not complain as a cultural trait, especially in more traditional, rural areas.

*National MoH cadre: I think it’s ... it’s a cultural issue. People do not really complain, unless it’s really intolerable.*

*Academic: And especially in the bush, these are people who are good, let us say. Who do not express themselves so much, who like the quiet life... So it’s not their culture to complain especially about teachers, about doctors, about all these state officials because they work in the ... so much in the bush.*

Complaining was seen as ill-mannered or done with the intention of spreading rumors, and thus not well regarded in society:

*CHW: [W]ell-behaved people do not talk too much, those who behave badly, even in public, [are the ones who] dare to speak up and denounce, especially if it is about drugs that they are made [to] buy at the pharmacy.*

*Fokontany (village) chief: In fact, if they speak, it is as if they are gossiping or denouncing their neighbor.*

### Low education levels impact interaction with authorities

Low education levels posed multiple complementary barriers to active participation during the NGO-led process:

a. Written expression

To enable anonymous complaints and suggestions that could feed into the consultative workshops as an additional source of information and thereby potentially counter the perceived fear of repercussions, the MoH made available a letter box at the health centers. However, many community members are unable to write letters due to their generally low education levels. Anonymous sharing of information via the internet also requires basic reading and writing skills, besides digital literacy:

*Regional government authority: The people who live in the bush are all people who are simple with their level of education. And the people who live in the district capitals, for example, that is different, the way they express themselves....*

b. Ability to actively take part in workshops.

The education level also had an impact on the capacity of community members and other workshop participants to engage meaningfully in technical discussions:

*Civil society representative: We have two types of people. There is the first who manages to understand but has difficulty in answering. The second one who cannot even understand and is easily lost.*

*CHW: [S]ince I have not studied much, I did not understand everything.*

c. French fluency as a pre-requisite for engagement.

Education levels are also linked to exposure to Madagascar’s other national language, French. Indeed, French was a barrier to participation cited by most interviewees from CSB and community level. The workshop leader spoke French but not Malagasy. Hence, plenary sessions were therefore held in both French and Malagasy, while group work was conducted in Malagasy only. Despite the plenary translations, many Malagasy-speaking Health Committee members and heads of CSB felt excluded and ashamed by the need for translation as they perceived themselves to thus not be at the same level as bilingual or French-speaking participants.

*CHW: I’d say… the use of foreign language was an obstacle. The lady [*ACF representative*] would speak and also the coordinator, they spoke a foreign language, and that stopped us short.*

*Head of a basic health center: The most difficult is understanding the French language. Most of the workshop was in French. We, the CSB, have terms that we do not understand that were new to us. French is not the mother tongue; there are others who just sat and listened [...] Next time, we would like it to be in both languages, the workshops, […] a few terms in French but it has to be in Malagasy. [...] it is better if the explanations are in Malagasy. […].*

Interestingly, government cadres in positions above the CSBs, who are generally bilingual, perceived the workshops to be held entirely in Malagasy, with minimal translation into French.

### Study objective (b): gain insight into which targeted efforts to engage communities might be successful in re-calibrating power relations

The following section will discuss measures undertaken to level out the power imbalances which prevented community members and other stakeholders from meaningfully contributing to the workshop (see Box 2) discussions.

### Smaller discussion groups without hierarchy elicited more frank discussion

Efforts were made to overcome the barriers preventing community representatives from actively participating in the workshops. Working groups were organized within the workshop, consisting of community members only, without hierarchies present. The aim was to enable an environment where community representatives could speak freely, without the fear of repercussions, among themselves and in their native language.

*District level government cadre: We try to adapt to this situation. We make a distribution of the group then. All participants are divided up. There are representatives of district services, there are representatives of NGOs. They are close to those communities, and that is why there is this approach; to overcome this difficulty between the bush and the city ...*

The need for more options to interact without the presence of social hierarchies was mentioned by several interviewees:

*District level government cadre: In my opinion, next time, if there will be another planning workshop you have to separate […]… in order to be able to express [their] ideas. [I]t will be very, very difficult for them to express in front of everyone.*

One good example of a targeted interaction with CHWs without the inclusion of social hierarchies played out in one of the district planning workshops. Noticing the reticence of CHWs to speak up, one central ministry representative took the initiative to take a group of them aside during a workshop break and speak with them separately. This scenario demonstrates the need to separate those at different levels of the social hierarchy to create a micro-space (in this case, the group of CHWs alone with the national ministry official) where the playing field is more level, to allow for meaningful voicing of concerns.

### Taking social participation activities into people’s own settings

Several interviewees mentioned that the meeting environment may have had an intimidating impact on participants who are used to meetings in their villages. CHWs as well as some CSB staff were not accustomed to being in a hotel and even less accustomed to being in front of a computer.

*Civil society representative: « dinidinika ambany kily » [debate under the tamarind tree] It’s more effective that way. This is the only way to get ideas out… It’s more effective especially for people in the southern region.*

Pre-meeting discussions with communities in their own environments tended to be more relaxed and open, indicating that keeping people in their own comfort zones, on their own territory, can be a good strategy to increase meaningful participation.

## Discussion

There is an evidence gap on how to *concretely* tackle power imbalances within the participatory process.

Our qualitative data shed light on the specific socio-cultural and economic barriers to participation within strategic health planning processes in two districts in Madagascar: fear of speaking up, the cultural value placed on keeping quiet, and low education levels. These challenges to participation have also been reported in other countries (7, 57), and are in many ways universal even if they may play out differently in different contexts (27, 58, 59). While these barriers are not new, they remain deeply entrenched and often overlooked in policy and practice.

In the two Malagasy districts studied, a clear power gradient exists between each level of the district health system: between community members and health facility staff who are perceived as having power over their health; between the CHW and health facility staff; and finally, between health facility staff and government officials higher up in the health system hierarchy who have higher education levels, are used to speaking in public, and are at ease with French. Despite the familiarity of these dynamics, policymakers still rarely take them into account when designing participatory processes.

This disequilibrium was strongly present within the Malagasy participatory space (NGO-led workshop) and led to less meaningful input from participants with less formal and informal power, i.e., community members and CHWs, in relation to those with more of it, i.e., CSB staff and government cadres from districts, regions, and national level. Consequently, views of those higher in the hierarchy prevailed, partly defeating the very purpose of setting up the bottom-up participatory planning workshop. This underscores the importance of designing participatory spaces that actively counterbalance power asymmetries—an approach that may sound simple but is still rarely implemented.

The unequal power gradient between the different stakeholders seemed to have influenced the issues which ended up being discussed during the participatory process as well, also a common phenomenon (60–62). For example, CHW concerns about their working conditions dominated workshop discussions rather than specific community needs or service delivery challenges. Community-level grievances, such as point-of-care payments or illicit drug sales, were not prioritized, though they clearly affect health-seeking behavior (63).

Higher education levels and technical skills, amongst many other factors, lead to a higher economic and socio-cultural status that is inherently connected to power and influence. The literature confirms the tendency of well-educated professionals to dominate participatory processes (28, 58, 64), either by using technical language or simply by their presence as participants may perceive them as more knowledgeable, thus–consciously or sub-consciously–giving their views more weight (65, 66).

Our study additionally points to three simple strategies which can be put in place to provide counter-vailing power to those with less voice due to socio-cultural and economic barriers. These strategies are not revolutionary, but their relevance lies in their practicality and the fact that they are still not widely adopted. Moreover, our findings contribute to the evidence base that such measures could work—addressing a persistent gap in the literature on how to concretely tackle power imbalances within participatory processes.

First, community members’ difficulties to fully grasp technical discussions can be counter – balanced by using the same language as those whose views and input are solicited – in our case, it not only means Malagasy rather than French in some situations but also reducing technical jargon and technical terms into more simple language (26, 60, 66).

Second, participatory space organizers can create smaller, if possible homogenous, groups for dialogue with participant groups whose voices are less heard. Putting participants initially with people whom they feel are similar to themselves can help participants feel safe and more at ease to speak up and give input. Indeed, Snow et al. recommend creating environments that normalize people’s life-context by surrounding them with people who have similar backgrounds and experiences (26). In the Malagasy setting, NGO staff went into communities for data collection prior to the workshop with the aim of discussing with community members and CHWs alone and in their own environment. Unfortunately, separate pre-workshop meetings with each of the participant groups were dropped due to time constraints. In the future, these are planned to be prioritized more.

The third strategy suggested through our data analysis is holding meetings away from the more intimidating and more official venues linked to official structures, and therefore, hierarchies. Bringing consultations to people’s own environment emphasizes the willingness of those in power to truly listen to what people want to say (7). It can also decrease the (opportunity) costs people face when participating in a consultative workshop, for example transportation costs, missing time from work, childcare, etc. (26).

The above-mentioned strategies require participatory space organizers to send, or work with, facilitators who speak the language of communities. Working in a different language requires adapting the participatory approach and reflecting on health issues from the viewpoint of communities expressed in their own words. Such fairly simple participatory process design features can thus put those with influence and official titles in a position where communities are the ones more comfortable and thus ‘powerful’, thereby contributing to levelling the playing field and enabling more meaningful engagement (7). These are not complex interventions—but they are powerful in their simplicity, and their consistent neglect in policy design makes their implementation even more urgent.

Our findings can be understood through the lens of the WHO social participation handbook, particularly its emphasis on voice, agency, and empowerment within participatory processes. In this perspective, the barriers observed—such as fear of speaking, language asymmetries, and hierarchical dynamics—reflect unequal distributions of power that constrain participants’ ability to exercise agency. The three strategies identified in this study can therefore be interpreted as practical design features that potentially help re-balance power within participatory spaces, by shifting communicative control, creating safer environments for expression, and embedding dialogue within participants’ own social contexts. In this sense, our study contributes to operationalizing how participatory processes can move closer to meaningful rather than symbolic participation.

## Limitations of the study

As a qualitative case study conducted in districts where an NGO-supported intervention was active, the findings are not statistically representative and may be influenced by selection and contextual biases, although they offer in-depth insights consistent with patterns reported in the wider literature.

Language was a study limitation for a sub-set of the interviews, i.e., those with the Malagasy-speaking heads of CSB and CHWs as the interviewer was francophone. The on-site translator conveyed summaries of the interviewee’s spoken word, resulting in some information being lost or lacking in detail. At the same time, this gave the possibility to study participants to express themselves in their mother tongue, an important factor as identified through our study.

Translation was thus both a methodological necessity and a subject of analysis in this study; while it may have introduced some loss of nuance during data collection, it also reflected broader language-related power asymmetries that were central to participants’ experiences.

In addition, many interviewees participated in some of the participatory workshops but not all of them. Others could not recall the process in detail. It was particularly difficult to find members of the central administration who were aware of the NGO-led participatory district planning process, the number of interviewees at the national level was therefore much smaller than the number of interviewees at the community and district level. Few community members were interviewed who were not community health workers, limiting the generalizability of findings.

## Conclusion

Despite the study’s limitations, our results mirror the literature on social participation in Madagascar, which identified social disconnection and a low level of education among poorer communities as limiting factors to participation (31). In addition, the literature also pointed towards traditionally ingrained notions of respect and awe of the hierarchy as an underlying factor for community members to stay silent during participatory exercises (33, 37).

Every society is inherently shaped by social, political, geographic, cultural and economic factors that create power imbalances between individuals as well as between population groups (67, 68). Consequently, this societal status-based disproportionate access to and influence on public policies is mirrored in the participatory micro-space and determines the success and sustainability of the participatory process (28), like it clearly did in the district participatory planning workshops in Madagascar, and many other similar participatory processes in other contexts (7).

Actively addressing structural inequalities and differences in societally attributed influence within the participatory space should be done with a holistic approach to levelling out stakeholder power asymmetries (7). The three strategies presented in this study are obviously small elements within that larger approach to fostering meaningful participation. They should be seen as complementary to larger investments in building government and civil society capacities to engage with each other, and complementary to efforts to build political will for participatory governance. However, the point we wish to make with these three strategies is that small participatory design features can go a long way to ensuring more meaningful participation.

More research is needed in settings like Madagascar where the literature base on community health is limited; workable intervention solutions which have been fine-tuned and adapted over time need solid documentation in order to support advocacy efforts towards the national government to invest more in this area to strengthen the nation’s health system.

## Data Availability

The raw data supporting the conclusions of this article will be made available by the authors, without undue reservation.

## Footnotes

1 From the 173 selected articles, 110 articles were published between 2018 and 2022.

## References

[1] Papanicolos I Rajan D Karanikolos M Soucat A Figueras J. Health System Performance Assessment: A Framework for Policy Analysis. Geneva: World Health Organization (2022). 37023239

[2] Fryatt R Bennett S Soucat A. Health sector governance: should we be investing more? BMJ Glob Health. (2017) 2:e000343. doi: 10.1136/bmjgh-2017-000343, 29225938 PMC5717939

[3] Francés F La Parra-Casado D. Participation as a driver of Health Equity. Copenhagen: WHO Regional Office for Europe (2019).

[4] Rajan D Koch K Mathurapote N Petrič V. Putting forward the value proposition: why prioritise social participation. Eurohealth. (2024) 30:19–23.

[5] Clark H Koonin J Cuevas Barron G. Social participation, universal health coverage and health security. Bull World Health Organ. (2021) 99:846–846A. doi: 10.2471/BLT.21.286554, 34866675 PMC8640689

[6] Rajan D Brocard E Poulussen C Koch K Nathan Limaro N Rohrer-Herold K . Beyond consultations and surveys: enhancing participatory governance in health systems. Eurohealth. (2022) 28:9–13.

[7] World Health Organization. Voice, Agency, Empowerment – Handbook on social Participation for Universal Health Coverage. Geneva: World Health Organization (2021).

[8] Rajan D Koch K. The health democracy deficit and COVID-19. Eurohealth. (2020) 26:26–8.

[9] Sitienei J Manderson L Nangami M. Community participation in the collaborative governance of primary health care facilities, Uasin Gishu County, Kenya. PLoS One. (2021) 16:e0248914. doi: 10.1371/journal.pone.0248914, 33788868 PMC8011762

[10] Masefield SC Msosa A Chinguwo FK Grugel J. Stakeholder engagement in the health policy process in a low income country: a qualitative study of stakeholder perceptions of the challenges to effective inclusion in Malawi. BMC Health Serv Res. (2021) 21:984. doi: 10.1186/s12913-021-07016-9, 34537033 PMC8449519

[11] Haricharan HJ Stuttaford M London L. The role of community participation in primary health care: practices of south African health committees. Prim Health Care Res Dev. (2021) 22:e31. doi: 10.1017/S146342362100027X, 34127167 PMC8220489

[12] Jacobs T George A. Between rhetoric and reality: learnings from youth participation in the adolescent and youth health policy in South Africa. Int J Health Policy Manag. (2022) 11:2927–39. doi: 10.34172/ijhpm.2022.6387, 35490263 PMC10105194

[13] Tefera BB Kibret MA Molla YB Kassie G Hailemichael A Abate T . The interaction of healthcare service quality and community-based health insurance in Ethiopia. PLoS One. (2021) 16:e0256132. doi: 10.1371/journal.pone.0256132, 34411148 PMC8376052

[14] Schaaf M Topp SM Ngulube M. From favours to entitlements: community voice and action and health service quality in Zambia. Health Policy Plan. (2017) 32:847–59. doi: 10.1093/heapol/czx024, 28369410 PMC5448457

[15] Kweku M Amu H Awolu A Adjuik M Ayanore MA Manu E . Community-based health planning and services plus programme in Ghana: a qualitative study with stakeholders in two systems learning districts on improving the implementation of primary health care. PLoS One. (2020) 15:e0226808. doi: 10.1371/journal.pone.0226808, 31914122 PMC6948830

[16] Iyanda OF Akinyemi OO. Our chairman is very efficient: community participation in the delivery of primary health care in Ibadan, Southwest Nigeria. Pan Afr Med J. (2017) 27:258. doi: 10.11604/pamj.2017.27.258.12892, 29187927 PMC5660304

[17] Mulumba M London L Nantaba J Ngwena C. Using health committees to promote community participation as a social determinant of the right to health: lessons from Uganda and South Africa. Health Hum Rights. (2018) 20:11–7. PMC6293345 30568398

[18] Kretchy IA Okoibhole LO Sanuade OA Jennings H Strachan DL Blandford A . Scoping review of community health participatory research projects in Ghana. Glob Health Action. (2022) 15:2122304. doi: 10.1080/16549716.2022.2122304, 36398761 PMC9677985

[19] Pare Toe L Dicko B Linga R Barry N Drabo M Sykes N . Operationalizing stakeholder engagement for gene drive research in malaria elimination in Africa-translating guidance into practice. Malar J. (2022) 21:225. doi: 10.1186/s12936-022-04241-3, 35870909 PMC9308116

[20] Sartas M van Asten P Schut M McCampbell M Awori M Muchunguzi P . Factors influencing participation dynamics in research for development interventions with multi-stakeholder platforms: a metric approach to studying stakeholder participation. PLoS One. (2019) 14:e0223044. doi: 10.1371/journal.pone.0223044, 31725717 PMC6855456

[21] Ward C Holmes G Stringer L. Perceived barriers to and drivers of community participation in protected-area governance. Conserv Biol. (2018) 32:437–46. doi: 10.1111/cobi.13000, 28833471

[22] Radhika D Tompkins EL Schreckenberg K. Forest ecosystem services derived by smallholder farmers in northwestern Madagascar: storm hazard mitigation and participation in forest management. Forest Policy Econ. (2017) 84:72–82. doi: 10.1016/j.forpol.2016.09.002

[23] Miakatra LS. Community participation and water supply in deprived areas of Madagascar. Field Act Sci Rep J Field Actions. (2014) 11:1–5.

[24] WSUP. Getting Communities Engaged in Water and Sanitation Projects: Participatory Design and Consumer Feedback. London: Water and Sanitation for the Urban Poor (2013).

[25] Nelson E Bloom G Shankland A. Accountability for Health Equity: Galvanising a Movement for Universal Health Coverage, IDS Bulletin 49.2. Brighton: IDS (2018). doi: 10.19088/1968-2018.127

[26] Snow ME Tweedie K Pederson A. Heard and valued: the development of a model to meaningfully engage marginalized populations in health services planning. BMC Health Serv Res. (2018) 18:181. doi: 10.1186/s12913-018-2969-1, 29544486 PMC5856315

[27] Frankish CJ Kwan B Ratner PA Wharf Higgins J Larsen C. Challenges of citizen participation in regional health authorities. Soc Sci Med. (2002) 54:1471–80. doi: 10.1016/s0277-9536(01)00135-6, 12061482

[28] Kapiriri L. Public participation in health planning and priority setting at the district level in Uganda. Health Policy Plan. (2003) 18:205–13. doi: 10.1093/heapol/czg025, 12740325

[29] Ministry of Health Madagascar. Politique Nationale de Santé Communautaire. Antananarivo: Ministry of Public Health Madagascar. (2017).

[30] Ministry of Health Madagascar. Stratégie Nationale sur la couverture santé universelle Madagascar. Antananarivo: Ministry of Public Health Madagascar. (2015).

[31] Razafindrakoto M Roubaud F Wachsberger JM. L’île mystérieuse: une approche d’économie politique de la trajectoire longue de Madagascar. Canad J Develop Stud. (2015) 36:397–415. doi: 10.1080/02255189.2015.1075870

[32] Razafindrakoto M. Élites, pouvoir et régulation à Madagascar: Une lecture de l’histoire à l’aune de l’économie politique. Afr Contemp. (2014) 251:25.

[33] Smith SM Dorward PT. Nationalised large-scale mining, trade unions and community representation: perspectives from northern Madagascar. Resour Policy. (2014) 40:31–41. doi: 10.1016/j.resourpol.2013.11.006

[34] Afrobarometer. Afrobarometer Briefing Paper. Report No.76. Accra: Afrobarometer (2009).

[35] Afrobarometer. Résumé des résultats Enquête d’Afrobarometer Round 10 à Madagascar. Accra: Afrobarometer (2024).

[36] Razafindrakoto M Roubaud F Wachsberger JM. L’énigme et le paradoxe: économie politique de Madagascar. Paris: Agence Française de Développement (2017).

[37] Smith SM Shepherd DD Dorward PT. Perspectives on community representation within the extractive industries transparency initiative: experiences from south-East Madagascar. Resour Policy. (2012) 37:241–50. doi: 10.1016/j.resourpol.2011.01.001

[38] du Chatelet A. Successes from the field: Using a bottom-up Approach to Strengthen Health Systems and Policies. Paris: Action Against Hunger (2017).

[39] Brousse S. L’expérience nutris: Capitalisation sur le renforcement du système de santé à Madagascar. Paris: Action contre la Faim (2018).

[40] Action contre la Faim. Renforcement des système de santé: Du diagnostique à la planification. Paris: Action contre la Faim (2016).

[41] Agalga S Alatinga KA Abiiro GA. Enablers and inhibitors of community participation in Ghana’s community-based health planning and services programme: a qualitative study in the Builsa north municipality. BMC Health Serv Res. (2022) 22:1468. doi: 10.1186/s12913-022-08869-4, 36461047 PMC9716718

[42] Simen-Kapeu A Lewycka S Ibe O Yeakpalah A Horace JM Ehounou G . Strengthening the community health program in Liberia: lessons learned from a health system approach to inform program design and better prepare for future shocks. J Glob Health. (2021) 11:07002. doi: 10.7189/jogh.11.07002, 33763217 PMC7956118

[43] Patton M. Qualitative Research & Evaluation Methods. 4th ed. Los Angeles, London, New Delhi, Singapore, Washington DC: SAGE (2014).

[44] Pope C Mays N Popay J. Synthesizing Qualitative and Quantitative Health Evidence: a guide to Methods. Maidenhead: Open Univ. Press (2007). p. 210.

[45] Bazeley P. Analysing qualitative data: more than ‘identifying themes’. Malays J Qual Res. (2009) 2:6–22.

[46] Leung L. Validity, reliability, and generalizability in qualitative research. J Fam Med Prim Care. (2015) 4:324–7. doi: 10.4103/2249-4863.161306, 26288766 PMC4535087

[47] Boateng MA Agyei-Baffour E Angel S Asare O Prempeh B Enemark U. Co-creation and prototyping of an intervention focusing on health literacy in management of malaria at community-level in Ghana. Res Involv Engagem. (2021) 7:55. doi: 10.1186/s40900-021-00302-0, 34353378 PMC8340491

[48] Gowelo S Meijer P Tizifa T Malenga T Mburu MM Kabaghe AN . Community participation in habitat management and Larviciding for the control of malaria vectors in southern Malawi. Am J Trop Med Hyg. (2023) 108:51–60. doi: 10.4269/ajtmh.21-1127, 36410320 PMC9833073

[49] Ng’ang’a PN Aduogo P Mutero CM. Strengthening community and stakeholder participation in the implementation of integrated vector management for malaria control in western Kenya: a case study. Malar J. (2021) 20:155. doi: 10.1186/s12936-021-03692-4, 33740983 PMC7977174

[50] Martini J Tijou Traoré A Mahieu C. Chronic patient as intermittent partner for policy-makers: the case of patient participation in the fight against diabetes and HIV/AIDS in Mali. BMC Public Health. (2019) 19:1179. doi: 10.1186/s12889-019-7453-2, 31455367 PMC6712700

[51] Kenworthy NJ. Participation, decentralisation and déjà vu: remaking democracy in response to AIDS? Glob Public Health. (2014) 9:25–42. doi: 10.1080/17441692.2013.879728, 24506667 PMC7986482

[52] Campbell C Williams B. Understanding the impact of a community-led HIV prevention program in South Africa: context, conceptual framework and methodology. Aust J Prim Health. (1999) 5:9–23.

[53] Ntshanga SP Ngcobo PS Mabaso MLH. Establishment of a community advisory board (CAB) for tuberculosis control and research in the Inanda, Ntuzuma and KwaMashu (INK) area of KwaZulu-Natal, South Africa. Health Policy. (2010) 95:211–5. doi: 10.1016/j.healthpol.2009.12.004, 20036434

[54] Silumbwe A Nkole T Munakampe MN Cordero JP Milford C Zulu JM . Facilitating community participation in family planning and contraceptive services provision and uptake: community and health provider perspectives. Reprod Health. (2020) 17:119. doi: 10.1186/s12978-020-00968-x, 32771028 PMC7414747

[55] Nkwonta CA Messias DKH. Male participation in reproductive health interventions in sub-Saharan Africa: a scoping review. Int Perspect Sex Reprod Health. (2019) 45:71–85. doi: 10.1363/45e8119, 31859670

[56] Silumbwe A Nkole T Munakampe MN Milford C Cordero JP Kriel Y . Community and health systems barriers and enablers to family planning and contraceptive services provision and use in Kabwe District, Zambia. BMC Health Serv Res. (2018) 18:390. doi: 10.1186/s12913-018-3136-4, 29855292 PMC5984360

[57] Williams J. "The politics of social change and the transition to democratic governance: community participation in post-apartheid South Africa". In: Pretorius J, editor. African Politics: Beyond the Third Wave of Democratisation. Cape Town: Juta (2008). p. 172–97.

[58] Gregory J Hartz-Karp J Watson R. Using deliberative techniques to engage the community in policy development. Aust N Z Health Policy. (2008) 5:16. doi: 10.1186/1743-8462-5-16 PMC2500036 18631378

[59] Ruano AL. The role of social participation in municipal-level health systems: the case of Palencia, Guatemala. Glob Health Action. (2013) 6:20786. doi: 10.3402/gha.v6i0.20786, 24028936 PMC3772320

[60] National Research Council. Public Participation in Environmental Assessment and Decision Making. Washington, D.C.: National Academies Press (2008).

[61] Siddiqui FR. Annotated bibliography on participatory consultations to help aid the inclusion of marginalized perspectives in setting policy agendas. Int J Equity Health. (2014) 13:124. doi: 10.1186/s12939-014-0124-0, 25532831 PMC4279799

[62] Martin GP. Representativeness, legitimacy and power in public involvement in health-service management. Soc Sci Med. (2008) 67:1757–65. doi: 10.1016/j.socscimed.2008.09.024, 18922611

[63] Chomi E Mujinja PG Enemark U Hansen K Kiwara A. Health care seeking behaviour and utilisation in a multiple health insurance system: does insurance affiliation matter? Int J Equity Health. (2014) 13:25. doi: 10.1186/1475-9276-13-25, 24645876 PMC3994926

[64] Rowe G Frewer LJ. Evaluating public-participation exercises: a research agenda. Sci Technol Hum Values. (2004) 29:512–56. doi: 10.1177/0162243903259197

[65] Organization for Economic Cooperation and Development. Challenge of Capacity Development. Working Towards Good Practice. Paris: OECD (2006).

[66] Lee SK Thompson SC Amorin-Woods D. One service, many voices: enhancing consumer participation in a primary health service for multicultural women. Qual Prim Care. (2009) 17:63–9. 19281676

[67] Bertana A. The role of power in community participation: relocation as climate change adaptation in Fiji. Environ Plann C Polit Space. (2020) 38:902–19. doi: 10.1177/2399654420909394

[68] Agarwal B. Participatory exclusions, community forestry, and gender: an analysis for South Asia and a conceptual framework. World Dev. (2001) 29:1623–48. doi: 10.1016/s0305-750x(01)00066-3

