# Supplementary material for: Community case study: overcoming barriers to social participation for improved local health governance in Madagascar
Source: Front Public Health. 2026 Sep 8;14:1778275. doi: 10.3389/fpubh.2026.1778275 (PMC13593836; doi:10.3389/fpubh.2026.1778275)
Supplement: Supplementary file 1 [file Table_1.DOCX]

**Questionnaire niveaux district et régional et OSC**

1. Pour commencer, pouvez-vous me parler des services de santé pour les communautés dans votre région ? Qu’est-ce qui marche bien, qu’est-ce qui marche moins bien, ou qu’est-ce qui manque ?

→ Les gens ont-ils des moyens de se plaindre ou un endroit où répondre à leurs préoccupations concernant ce qui ne fonctionne pas bien dans votre communauté ?

1. Pouvez-vous nous parler de votre OSC et du travail qu’elle fait avec les communautés dans le domaine de la santé pour connaitre leurs besoins ?
2. Pouvez-vous me parler des COSAN/COGES dans votre district/ région. Quel est leur rôle dans la communauté ?

Qui peut faire partie d’un COSAN/COGES et comment ?

Quel est votre opinion du fonctionnement de ces comités ?

3. Expliquez-moi comment vous travaillez/collaborez avec ces comités, ainsi qu’avec les CSB?
   a. Quand OSC interviewées : quelle est votre interaction avec les autorités sanitaires et administratives du district et de la Région
   b. Quand DS et Région interviewés : quelle est votre interaction avec les OSC locales et régionales
4. Selon votre expérience, est ce que les gens ont l’habitude de s’exprimer en général à Madagascar/ dans la vie du village/Fokontany?
5. Comment les COSAN/COGES dans votre région/district, ont pu construire une vision commune sur la santé ?

Qui peut participer, et comment ?

(Probe : Est-ce que tous les membres de la communauté ont la possibilité de participer ? Les hommes et les femmes également ? Peut-être des groupes vulnérables spécifiques (par exemple, handicapés) ?

6. Quels mécanismes et quels espaces ont été utilisés pour que les membres de la communauté puissent donner leur avis sur les services de soins disponibles et sur les activités en santé à mettre en place?
7. Quels sont les processus en place pour faire entendre la voix des communautés à travers les différents niveaux du système de santé ?
   a. Quel est votre rôle en tant que OSC pour faire entendre la voix des communautés ?
8. Pouvez-vous me parler du processus de planification RSS de votre point de vue, de votre expérience ? Identification des activités prioritaires, services de soins disponibles, aider le CSB à ajuster ces services

Quel est votre rôle dans la planification RSS ?

Comment à travers votre rôle pouvez-vous faciliter l’implication des communautés dans ce processus

9. Quel est/était le rôle des communautés au cours du processus de l’élaboration de la feuille de route RSS/du Plan Annuel de Travail (PTA) du District. *(Ici il será nécessaire de leur rappeler que cette feuille de route a été développée lors de l’atelier de planification où les activités prioritaires ont été identifiées)* (liée au RSS) / le rôle de votre OSC ? Quel est votre rôle pour faciliter la participation des communautes?
10. De votre point de vue, dans quelle mesure la situation des communautés a été pris en considération lors du processus de planification RSS ? Exemples ?
11. Comment les phases de diagnostic et de planification du RSS ont-t-elles permis de faciliter la remontée des besoins spécifiques des populations/ communautés ?
12. Comment le RSS vous a-t-il aidé à comprendre les besoins des communautés et à les prendre en compte dans la planification RSS?
13. De votre point de vue, quelles ont été les valeurs ajoutées du processus RSS pour les communautés/ districts ?

Probe : comité de pilotage, ateliers de diagnostic et de planification

14. Tulear I : Les activités mises en œuvre depuis le début du RSS (2016), reflètent-elles ce que les communautés ont exprimé lors de la planification ? Exemples ?

Tulear II : Les activités planifiées reflètent-elles ce que les communautés ont exprimé lors de l’atelier de planification ? Exemples ?

15. Depuis l’approche RSS, les communautés ont-elles été plus impliquées/actives dans le processus de planification sanitaire qu’avant ?
16. Quels sont les difficultés que les communautés ont rencontrés dans la participation au processus RSS?

Pouvez-vous partager des exemples quand les communautés, vouliez participer mais ne pouviez pas ?

17. Pouvez-vous donner des exemples de propositions ou solutions que les communautés ont faites lors de la planification ? Comment s'est déroulé le processus pour trouver ces solutions? Comment étaient-elles reçues ?

Etes-vous au courant des suggestions qui n’étaient pas pris en compte ?

18. Êtes-vous au courant des suggestions spécifiques que les communautés ont exprimées et qui sont maintenant intégrées dans la planification RSS ? Lesquelles ?
19. De quel type de préparation ont les communautés eu besoin pour participer ? Qui les a soutenus dans leur participation et comment ?
20. Parlez-moi des facteurs qui ont facilité la participation des communautés ? Et des OSC ? Comment le ministère de la santé facilite la participation des communautes?
21. OSC : A travers quels mécanismes les messages de plaidoyer ont-ils pu être porté (plateformes, espaces de discussions, etc.) ? Quels mécanismes se sont révélés plus efficaces que les autres ?

Quel est votre rôle ?

22. Pouvez-vous me parler des cadres de concertations actuels (comités liés à la santé, rencontres, etc.) ? Et quel est votre rôle dans le cadre de concertation ?
23. Parlez-moi du rôle des autorités sanitaires dans le processus de planification RSS.
24. Quel rôle jouent les autorités sanitaires afin d’impliquer (ou pas) la communauté ?

Comment ?

25. Pensez-vous que les mécanismes actuels permettent de bien prendre en compte les besoins/doléances de la communauté dans les activités des CSB, du DS et de la Région ? Le RSS a-t-il facilité la mise en œuvre de ces mécanismes ?
26. Le RSS vous a-t-il aidé à analyser la façon dont les communautés sont prises en compte dans la planification ?
    a. Pour DS et Région : Avez-vous fait des ajustements sur la façon de faire la planification au niveau District/Région par la suite ?
    b. Pour OSC : Avez-vous revu votre stratégie d’action pour appuyer les communautés par la suite ?

c. est-ce-que le RSS vous a permis de vous rendre compte que les communautés n’étaient pas bien/ou au contraire bien prises en compte dans la planification sanitaire?

27. Pour OSC : Comment le RSS vous-a-t-il aidé dans votre travail avec les communautés sur les thématiques de santé (alignement avec d’autres OSC/autorités, plan d’action commun, espaces de discussions, etc.) ?
