# Supplementary material for: Community case study: overcoming barriers to social participation for improved local health governance in Madagascar
Source: Front Public Health. 2026 Sep 8;14:1778275. doi: 10.3389/fpubh.2026.1778275 (PMC13593836; doi:10.3389/fpubh.2026.1778275)
Supplement: Supplementary file 2 [file Table_2.DOCX]

**Questionnaire COSAN (commune et Fokontany) et Chef CSB**

1. Pour commencer, pouvez-vous me parler des services de santé dans votre communauté/ dans votre village.
   i. Qu’est-ce qui marche bien, qu’est-ce qui marche moins bien, ou qu’est-ce qui manque ?
   ii. **→Les gens ont-ils des moyens de se plaindre ou un endroit où répondre à leurs préoccupations concernant ce qui ne fonctionne pas bien dans votre communauté en terme de santé?**
2. Est-ce que les gens ont l’habitude de s’exprimer dans le village ou la communauté ?
3. Pouvez-vous me parler de votre COSAN F/COSAN C. Quel est votre rôle dans votre communauté ?
   i. Qui peut faire partie d’un COSAN F/COSAN C et comment ?
   ii. Quel est votre opinion du fonctionnement de ces comités ?

4. Pouvez-vous nous expliquer comment vous participez à faire la conexión/le pont entre la communauté et le CSB?

5. Dans votre COSAN F/COSAN C/CSB, comment avez-vous pu construire une vision commune par rapport à la santé ? Comment vous avez décidé ensemble ce que vous voulez pour votre commune ?
   a. Exemple : Palu – rôle de COSAN pour la prise en charge ?
      i. Qui peut participer, et comment ?
      ii. (Probe : Est-ce que tous les membres de la communauté ont la possibilité de participer ? Les hommes et les femmes également ? Peut-être des groupes vulnérables spécifiques (par exemple, handicapés) ?
6. Quels mécanismes et quels espaces sont utilisés pour que les membres de la communauté puissent donner leur avis ?
7. Quels sont les processus en place pour faire entendre votre voix, celle de votre communauté, à travers les différents niveaux du système de santé. – Retour ?
   i. Réunions régulières
8. Pouvez-vous me parler du processus de planification sanitaire ;  de votre point de vue, de votre expérience ? Identification des activités prioritaires, services de soins disponibles, aider le CSB à ajuster ces services, etc.
   i. Quel est votre rôle dans cette planification ?
9. Pouvez-vous me parler des différents mécanismes que vous pouvez utiliser en tant que membre de la communauté pour vous engager dans la planification des activités en santé du CSB et du District Sanitaire?
10. Parlez-moi du rôle des autorités sanitaires dans le processus de planification sanitaire.
11. Parlez-moi de l’approche RSS qu’ACF et ASOS ont mis en place dans votre communauté.

a. Quel est/était le rôle de votre communauté/COSAN/CSB au cours de l'élaboration de la feuille de route RSS/du Plan Annuel de Travail (PTA) du CSB. *(Ici il será nécessaire de leur rappeler que cette feuille de route a été développée lors de l’atelier de planification où les activités prioritaires ont été identifiées)* (liée au RSS) / le rôle de votre OSC ?

12. De votre point de vue, comment votre situation (en tant que communauté) / la situation des communautés a été pris en considération lors du processus RSS ? Exemple ?
    a. Est-ce que c’était différent que la planification PTA ? Comment ?
13. Comment les phases de diagnostic et de planification du RSS ont-t-elles permis de faciliter la remontée des besoins spécifiques des populations de votre communauté ?
14. Pouvez-vous donner des exemples de propositions ou solutions que vous avez faites lors de la planification ? Comment s'est déroulé le processus pour trouver ces solutions ? Comment étaient-elles reçues ?
15. Quelles ont été les valeurs ajoutées du processus RSS pour votre communauté ?
    i. Probe : comité de pilotage, ateliers de diagnostic et de planification
16. Tulear I : Les activités mises en œuvre depuis le début du RSS (2016), reflètent-elles ce que les communautés ont exprimé lors de la planification ? Exemples ?
    a. (Lors des années qui ont suivies) l’approche RSS, votre communauté a-t-elle été plus impliquée/active dans l’identification des priorités ou des problèmes par rapport aux services de santé ?
    b. Tulear II : Les activités planifiées reflètent-elles ce que les communautés ont exprimé lors de l’atelier de planification ? Exemples ?

17. Quelles sont les difficultés que vous avez rencontré lors du processus RSS en tant que communauté?

i. Pouvez-vous partager des exemples où vous, en tant que communauté, vouliez participer mais ne pouviez pas ?
ii. Avez-vous des idées comment il aurait fallu faire ?
iii. Y-a-t-il des suggestions qui n’étaient pas pris en compte ?

18. Êtes-vous au courant des suggestions spécifiques que vous avez exprimées en tant que communauté et qui sont maintenant intégrées dans le PTA du district/ la feuille de route ? Quelles suggestions ?
19. De quel type de préparation aviez-vous eu besoin pour participer en tant que communauté ? Qui vous a soutenus dans votre participation et comment ?
20. Parlez-moi des facteurs qui ont facilité votre participation en tant que communauté dans le RSS?
21. A travers quels mécanismes les messages de plaidoyer/revendication ont-ils pu être porté (plateformes, espaces de discussions, associations locales, etc.) ? Quels mécanismes se sont révélés plus efficaces que les autres ?
22. Quel rôle jouent les OSC pour faire entendre la voix de votre communauté ? Que pensez-vous de ce processus / mécanisme ?
23. Quel rôle jouent les autorités sanitaires afin d’impliquer (ou pas) la communauté ?
    i. Comment ?
