# Supplementary material for: Community case study: overcoming barriers to social participation for improved local health governance in Madagascar
Source: Front Public Health. 2026 Sep 8;14:1778275. doi: 10.3389/fpubh.2026.1778275 (PMC13593836; doi:10.3389/fpubh.2026.1778275)
Supplement: Supplementary file 3 [file Table_3.DOCX]

**Questionnaire niveau central**

1. Pour commencer, pouvez-vous me parler des services de santé pour les communautés dans la région Atsimo-Andrefana ?

Qu’est-ce qui marche bien, qu’est-ce qui marche moins bien, ou qu’est-ce qui manque ?

Les gens ont-ils des moyens de se plaindre ou un endroit où répondre à leurs préoccupations concernant ce qui ne fonctionne pas bien dans la communauté ?

2. Selon votre expérience, est-ce que les gens à Madagascar ont l’habitude de s’exprimer ?
3. Pouvez-vous me parler des COSAN dans cette région. Selon vous, quel est leur rôle dans la communauté ?
4. Selon vous, est-ce que les COSAN sont bien placés pour exprimer les besoins de la communauté en termes de santé ? Comment ? Pourquoi (pas) ?
5. Quelles plateformes et quels espaces sont utilisés pour que les membres de la communauté puissent donner leur avis ?
6. Quels facteurs pourraient empêcher les gens de la communauté de donner leur avis ?
7. Quels sont les processus en place pour faire entendre la voix des communautés à travers les différents niveaux du système de santé.
8. Quels types de remontées arrivent au niveau central ? Par quels mécanismes ?
9. Les COSAN/COGES/CSB dans la région Atsimo-Andrefana, comment ont-elles pu réaliser une vision commune par rapport à la santé ?
10. Pouvez-vous me parler du processus de planification sanitaire de votre point de vue, de votre expérience. Comment le niveau central assure-t-il la prise en compte de la situation des communautés dans l’élaboration des politiques de santé/planification en santé ?
11. Dans le Plan National de la Santé Communautaire, la participation communautaire joue un rôle central.
    a. Selon vous, qu’est-ce que ceci signifie ?
    b. Comment ceci fonctionne actuellement ? Exemples ?
12. Pouvez-vous me parler des différents stades et mécanismes que les communautés peuvent utiliser pour s’engager dans la planification sanitaire aux différents niveaux du système de santé ?
13. Pouvez-vous partager des exemples quand les communautés, vouliez participer mais ne pouviez pas ?
14. Quel rôle jouent les OSC/votre OSC pour faire entendre la voix des communautés ? Que pensez-vous de ce processus / mécanisme ?
15. Parlez-moi du rôle des autorités sanitaires dans le processus de planification sanitaire au niveau district ?
16. Quel rôle jouent les autorités sanitaires afin d’impliquer (ou pas) la communauté ?
17. **Parlez-moi de l’approche RSS mise en œuvre par ACF et ASOS :** Quel est/était le rôle des communautés à Atsimo-Andrefana au cours de l'élaboration de la feuille de route RSS, utilisée pour contribuer au Plan de Travail Annuel des Districts ?
18. De votre point de vu, dans quelle mesure la situation des communautés a été pris en considération lors du processus de planification sanitaire ? Exemples ?
19. Comment la phase de diagnostic et de planification a-t-elle permis de faciliter la remontée des besoins spécifiques des populations ? Parlez-moi de ce processus et des différents acteurs.
20. Les activités mises en œuvre depuis le début du RSS (2016), reflètent-elles ce que les communautés ont exprimé lors de la planification ? Exemples ?
21. Lors des années qui ont suivies l’approche RSS, les communautés ont-elles été plus impliquées/actives dans le processus de planification sanitaire qu’avant ?
22. Quels sont les facteurs limitants liés au processus RSS qui n’ont pas favorisé l’implication des communautés dans le processus de planification sanitaire ?
23. Êtes-vous au courant des suggestions spécifiques que les communautés ont exprimées et qui sont maintenant intégrées dans la stratégie nationale ? Lesquelles ?
24. De quel type de préparation ont les communautés eu besoin pour participer au processus de planification ?
25. Parlez-moi des facteurs qui ont facilité la participation des communautés ?

***PNSC :***

*Appropriation de la santé par la communauté : La communauté devrait être convaincue que la santé est la base de tout développement. Elle doit prendre conscience d’elle-même et de ses problèmes de santé, identifier elle-même les solutions locales et s’impliquer dans la surveillance de la santé publique. Elle est ainsi non seulement bénéficiaire des programmes de santé exécutés au niveau communautaire, mais prend également et activement part à la mise en œuvre des activités de promotion, de prévention, de surveillance, d’urgence, de réadaptation et curatives y afférentes pour une meilleure santé.*

*Planification ascendante : Chaque communauté a sa spécificité et les initiatives locales seront ainsi privilégiées, tout en tirant profit de la riche diversité socio-culturelle de la société malgache. Les problèmes identifiés localement seront résolus à travers des stratégies de proximité appropriées et élaborées par tous les acteurs de la santé communautaire, en partenariat avec les parties prenantes, et intégrées à la planification du Centre de Santé de Base (CSB) de rattachement pour être mises en œuvre. PNSC 2016*
